# Saddle‐shaped rotating knee prosthesis outperforms low contact stress knee: A short‐term retrospective comparative study

**DOI:** 10.1002/jeo2.70072

**Published:** 2024-12-02

**Authors:** Masafumi Itoh, Junya Itou, Umito Kuwashima, Ken Okazaki

**Affiliations:** ^1^ Department of Orthopedic Surgery Tokyo Women's Medical University Shinjuku Tokyo Japan

**Keywords:** low contact stress, patellar alignment, patellar score, patellar‐nonresurfacing, rotating concave–convex, rotating platform

## Abstract

**Purpose:**

This retrospective comparative study aimed to compare the native patellar alignment and clinical outcomes of the Rotating Concave–Convex (ROCC) knee, which features a saddle‐shaped rotating platform (RP) insert and a deep trochlea, versus the low contact stress (LCS) knee, which has favourable long‐term outcomes and features an anatomically shaped trochlea and a cruciate‐sacrificing RP insert. We hypothesized that the deeper trochlea of the ROCC would further stabilize the native patella, resulting in superior clinical outcomes compared to LCS‐RP.

**Methods:**

Consecutive patients who underwent patellar‐nonresurfacing primary total knee arthroplasty (TKA) using ROCC or LCS‐RP were retrospectively reviewed. Patients with over 1‐year post‐TKA follow‐up were included. Patients with neurologic disorders affecting knee function or additional ipsilateral knee surgery before evaluation were excluded. Patellar alignment was evaluated using the patella tilting angle (PTA), patella shift (PS), and the ratio of patella fitting depth into the trochlea (*F*) on axial radiographs. Primary and secondary outcomes were evaluated using the patellar score assessing patellofemoral function and Knee Injury and Osteoarthritis Outcome Score. Multiple regression analyses were performed with primary and secondary outcomes as dependent variables.

**Results:**

The analysis included 113 ROCC knees and 94 LCS‐RP knees (median follow‐up: 25 months, follow‐up rate: 92.3%). For ROCC and LCS‐RP, respectively, the mean PTA was 0.3 (3.2)° and 4.3 (2.9)°; PS was 0.5 (1.8) and 2.0 (2.5) mm; and *F* was 29.6 (8.1)% and 21.4 (6.5)% (all *p* < 0.001). On multivariate analysis, ROCC positively affected both primary and secondary outcomes (*p* = 0.004 and 0.0003–0.02, respectively).

**Conclusion:**

At short‐term follow‐up, ROCC stabilized the patella further horizontally, centrally, and deeply into the trochlea, thus outperforming LCS‐RP clinically. Orthopaedic surgeons should consider these potential advantages when selecting TKA models, especially those featuring cruciate‐sacrificing RP mechanisms in patellar‐nonresurfacing procedures.

**Level of Evidence:**

III. Retrospective comparative study.

## Graphical abstract

Saddle‐shaped rotating knee prosthesis significantly stabilized the native patella horizontally, centrally, and deeply, outperforming the low contact stress knee in short‐term clinical outcomes.

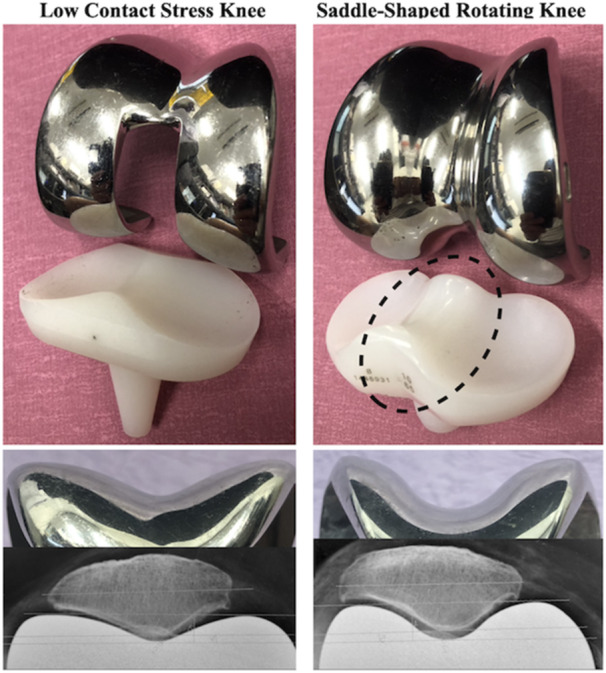

**Abbreviations**
ACH: anterior condyle height
ADL: activities of daily living
BMI: body mass index
*F*
: fitting (depth of the patella into the trochlea)
HKA: hip–knee–ankle (angle)
ICC: intraclass correlation coefficient
KOOS: knee injury and osteoarthritis outcome score
LCS: low contact stress
PCL: posterior cruciate ligament
PROM: patient‐reported outcome measure
PS: patella shift
PTA: patella tilting angle
ROCC: rotating concave–convex
RP: rotating platform
SEA: surgical epicondylar axis
TKA: total knee arthroplasty
UCLA: University of California‐Los Angeles

## INTRODUCTION

**Figure 1:**
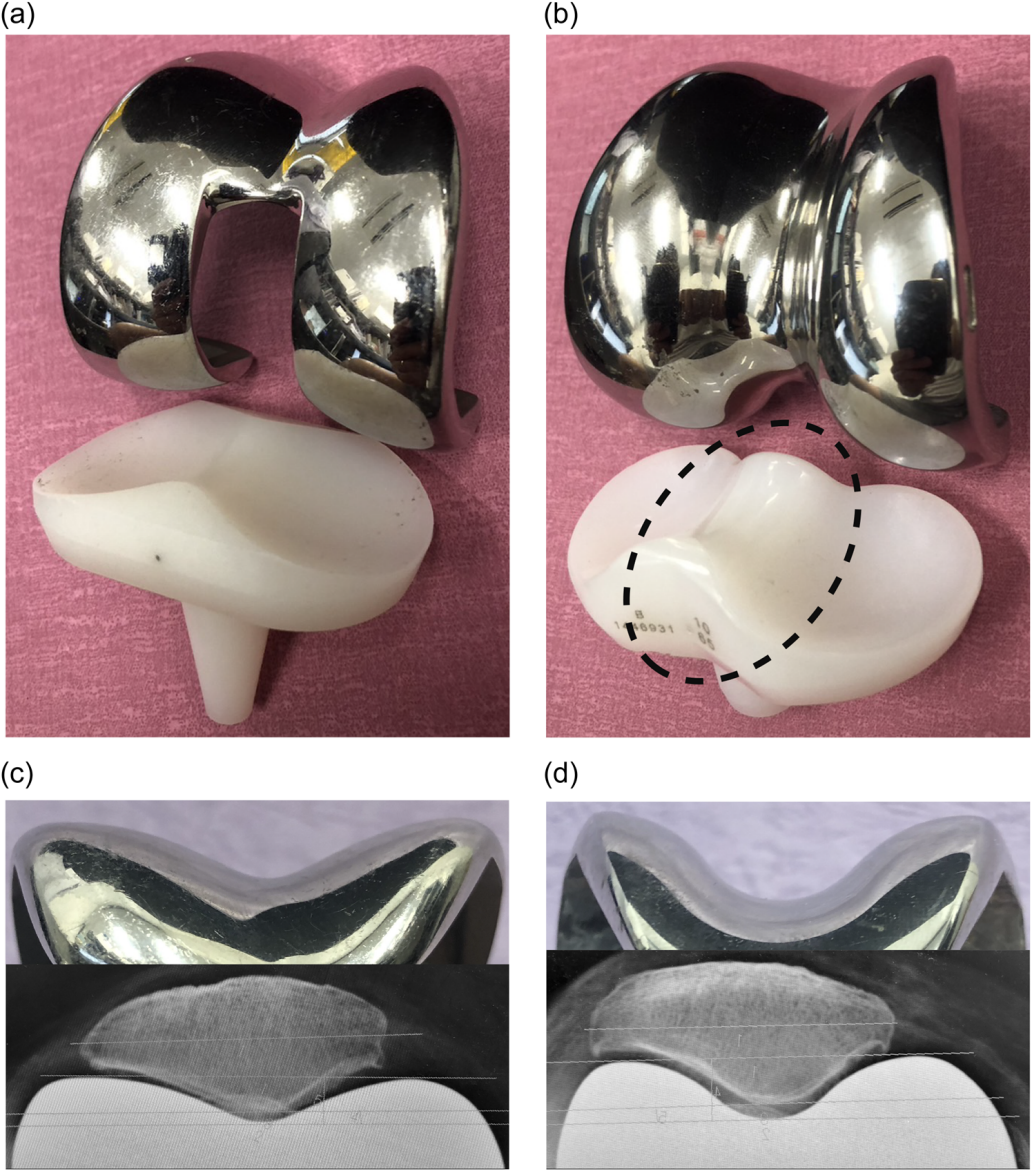
Comparison of the appearance of LCS‐RP with that of ROCC, shown by actual implants and axial radiographs. (a) Appearance of LCS‐RP: femoral component with a deep anatomical trochlear groove and an RP insert with a high anterior lip. (b) Appearance of ROCC: RP inserts with a hyperbolic paraboloid (saddle‐shaped) prominence (dotted ellipse) and femoral component with a deep trochlea that receives the saddle‐shaped prominence. (c) Femoral trochlea of LCS‐RP: the native patella fits into the anatomically shaped femoral trochlea of LCS‐RP. (d) Femoral trochlea of ROCC: the native patella fits more deeply into the deeper femoral trochlea of ROCC than that of LCS‐RP. LCS, low contact stress; ROCC, rotating concave–convex; RP, rotating platform.

Replacement or retention of the patella during total knee arthroplasty (TKA) remains a point of discussion [8, 11, 12]. TKA with patellar resurfacing has been associated with a lower risk of anterior knee pain [12]. However, patellar resurfacing is associated with rare but major complications, including fractures or osteonecrosis of the native patella and breakage or loosening of the patellar component [37]. To avoid such complications, some surgeons prefer non‐resurfacing options, which may include patellaplasty (osteophyte removal, circumferential denervation and lateral facetectomy) [16]. Moreover, femoral components with a deep anatomical trochlea groove have been shown to reduce anterior knee pain with the native patella [12], while rotating platform (RP) inserts can reportedly improve patellofemoral contact stress [36]. The rotational freedom of the posterior cruciate ligament (PCL)‐sacrificing RP insert allows it to self‐align with the femoral component, which optimizes patellar tracking and distributes contact stresses more evenly across the patellofemoral joint, and the lack of a post‐cam mechanism, which could cause patellar clunk syndrome, potentially reduces the risk of anterior knee pain [17, 36]. The low contact stress (LCS)‐RP (DePuy) features a deep anatomical trochlea groove and PCL‐sacrificing RP insert (Figure 1). LCS‐RP has excellent mid‐to‐long‐term clinical results [18, 29], potentially demonstrating the effectiveness of this mechanism in patellar‐nonresurfacing TKA.

Rotating concave–convex (ROCC) TKA (Zimmer Biomet) has demonstrated excellent mid‐to‐long‐term clinical outcomes [3, 4], and was developed based on the same fundamental design concept as LCS‐RP. The key feature of ROCC is its saddle‐shaped insert, which provides high conformity and stability during deep knee flexion, as well as a deeper trochlea to accommodate the insert [3, 5, 23] (Figure 1). To date, no studies have compared the clinical outcomes between ROCC and LCS‐RP. This comparison is crucial for understanding how different PCL‐sacrificing RP designs affect native patellar stability and patient outcomes, potentially influencing future implant selection. The aim of this retrospective comparative study was to compare the post‐operative native patellar alignment and clinical outcomes between ROCC and LCS‐RP. We hypothesized that the deeper trochlea of the ROCC further stabilizes the native patella, resulting in superior clinical outcomes than LCS‐RP, which has been reported to be unaffected by advanced patellofemoral arthritis or patellar morphology and would serve as an ideal benchmark due to its favourable long‐term outcomes in patellar‐nonresurfacing cases [18, 20, 29, 32].

## MATERIALS AND METHODS

### Ethics

This retrospective cohort study was approved by the Ethics Committee of Tokyo Women's Medical University (approval number: 4578) and conducted in accordance with the ethical standards of the Declaration of Helsinki. Informed consent was obtained from all patients.

### Study design and patient selection

**Figure 2:**
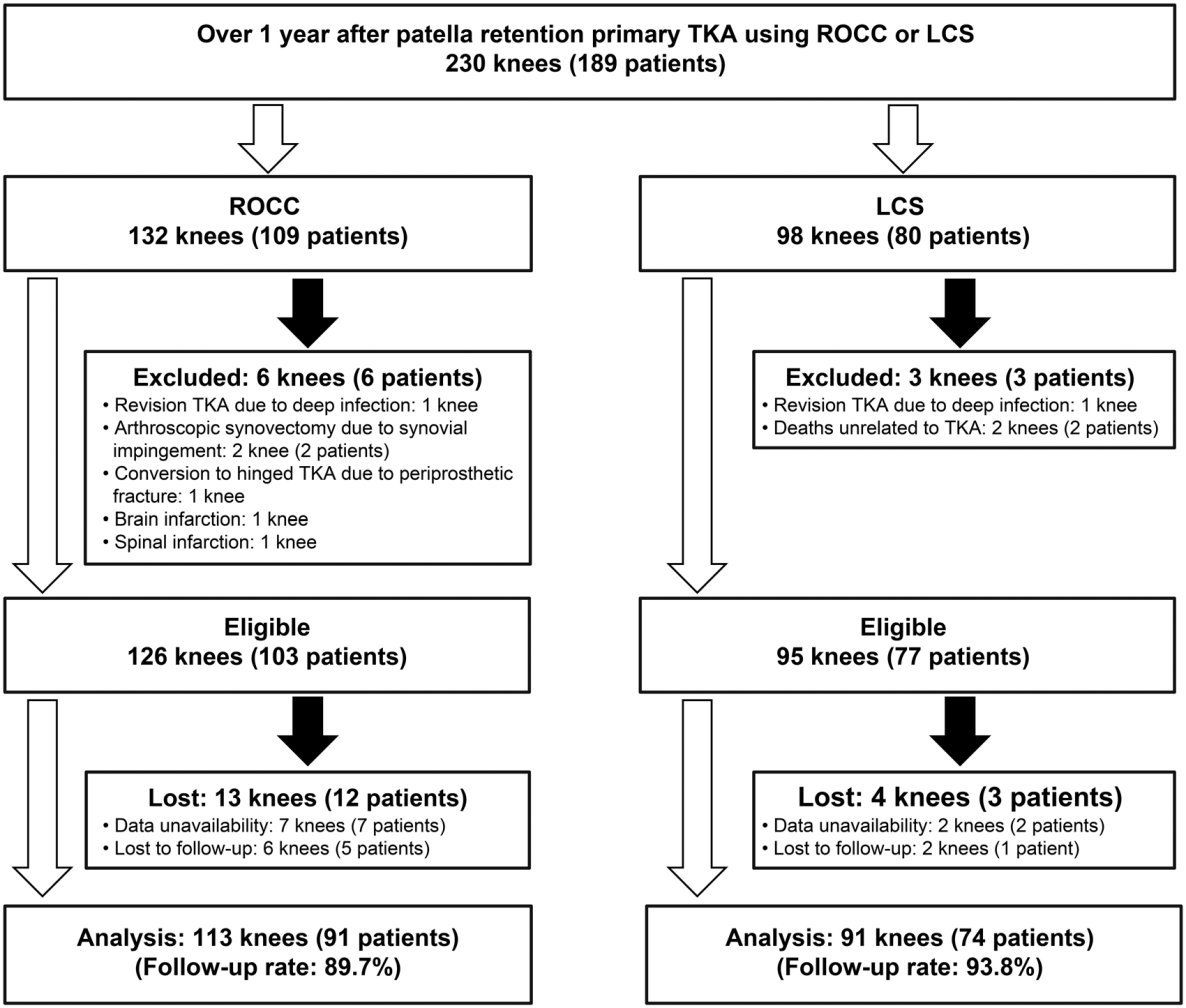
Flow diagram of this retrospective study. LCS, low contact stress; ROCC, rotating concave–convex; TKA, total knee arthroplasty.

The study retrospectively reviewed 230 consecutive knees from 189 patients who underwent primary patellar‐nonresurfacing TKA between September 2012 and September 2021 at our hospital using ROCC or LCS (ROCC: 132 knees from 109 patients, LCS: 98 knees from 80 patients), indicated for osteoarthritis, osteonecrosis, and rheumatoid arthritis without restrictions for the severity of patellar articular degeneration and contraindicated for collateral ligament dysfunction and knee infection. Patellar dysplasia and posttraumatic osteoarthritis were not present in the 230 knees. Inclusion criteria were: (1) patients with over 1 year of post‐TKA follow‐up, and (2) patients with complete data sets. Exclusion criteria were: (1) patients who developed neurological disorders significantly affecting knee function, and (2) patients who underwent additional ipsilateral knee surgery before evaluation. The surgeon's preference determined implant choice. Figure 2 shows the flow diagram of this study. No patient in either group underwent secondary patellar resurfacing.

### Surgical procedure and post‐operative protocol

Both TKA systems were performed by four surgeons experienced in knee arthroplasty, using the following technique. Subvastus arthrotomy was performed without dissecting the superficial layers of the medial collateral ligament, and both cruciate ligaments were resected. The tibia was cut 10 mm distal to the most proximal point of the articular surface perpendicular to the tibial axis in the coronal plane with a posterior slope of 0° for ROCC and 7° for LCS in the sagittal plane (ROCC insert contains 7° and LCS‐RP insert contains 0° of posterior slope, respectively). In both groups, the distal femur was cut, aiming for mechanical alignment, and the size and rotation of the femoral component were determined using the gap balancing technique [4, 14]. Regardless of patellar degeneration severity, all the patellae were retained, and osteophyte resection was performed without cauterizing the patellar rim [18]. No case required lateral release. Insert thickness was determined to achieve full extension and medial stability in both extension and flexion. Cementless implants in all cases were used, except for one ROCC knee (0.9%) due to poor bone quality. Both groups received single‐protocol pain management post‐operatively and underwent rehabilitation without restrictions on activities of daily living.

### Radiological assessment

**Figure 3:**
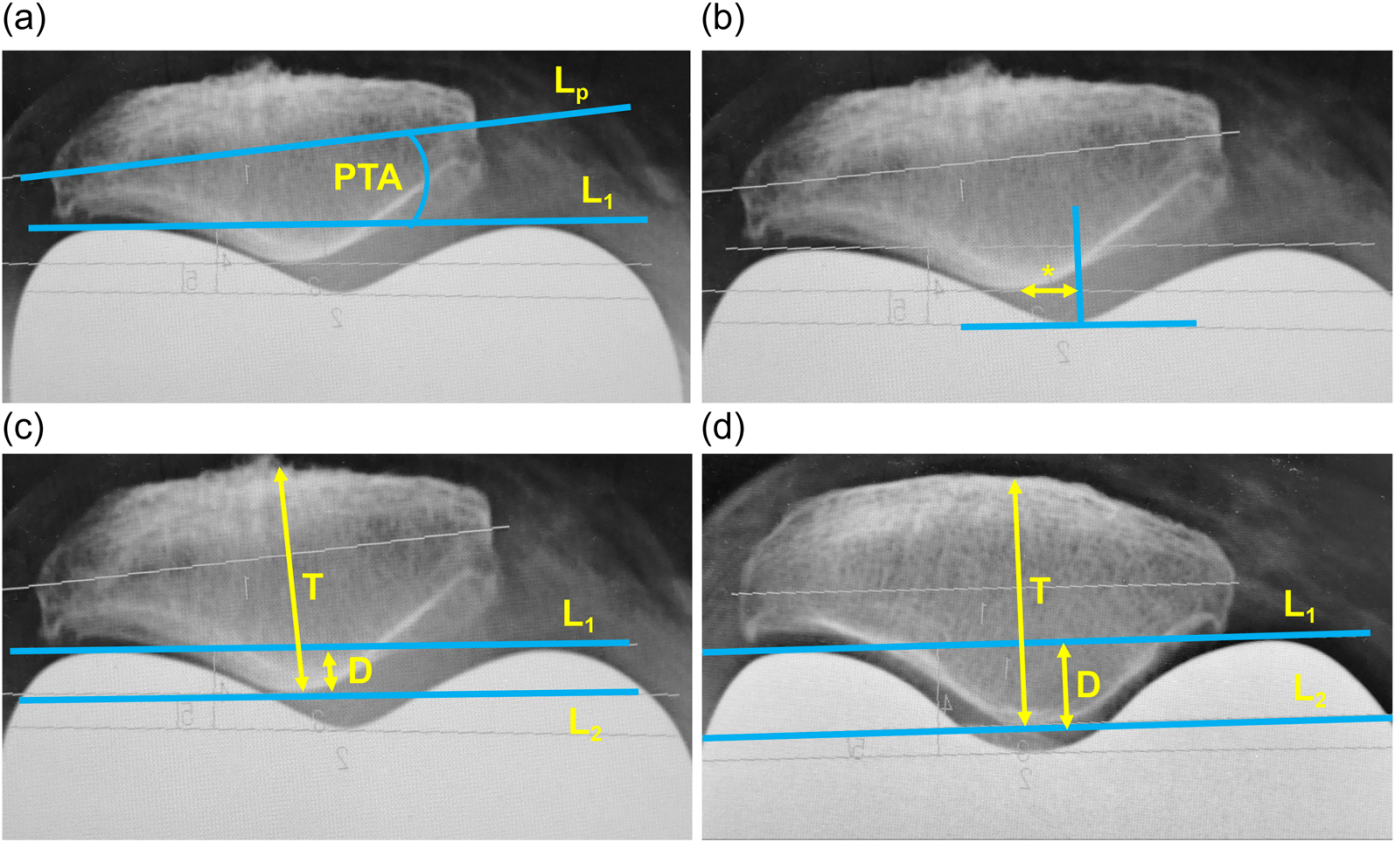
Explanation on PTA, PS and *F* which is defined as an indicator for the fitting depth of the patella into the femoral trochlea and expressed by the following equation: *F* (%) = (*D*/*T*) × 100. (a) PTA is the angle (°) between the transverse axis of the patella (*L*_p_) and the anterior intercondylar line (*L*_1_), considered positive when the patella tilted laterally. (b) PS is the distance (mm) between the central ridge of the patella and the deepest point of the femoral trochlea (asterisk), considered positive when the patella tilted laterally. (c) *F* is low when the fitting is shallow (LCS; *F* = 18%). (d) *F* is large when the fitting is deep (ROCC: *F* = 34%). *D*, partial patellar thickness located below *L*_1_; *L*_1_, anterior intercondylar line; *L*_2_, line parallel to the *L*_1_ and passing through the central ridge of the patella; *L*_p,_ transverse axis of the patella; LCS, low contact stress; PS, patella shift; PTA, patella tilting angle; ROCC, rotating concave–convex; *T*, total patellar thickness.

**Figure 4:**
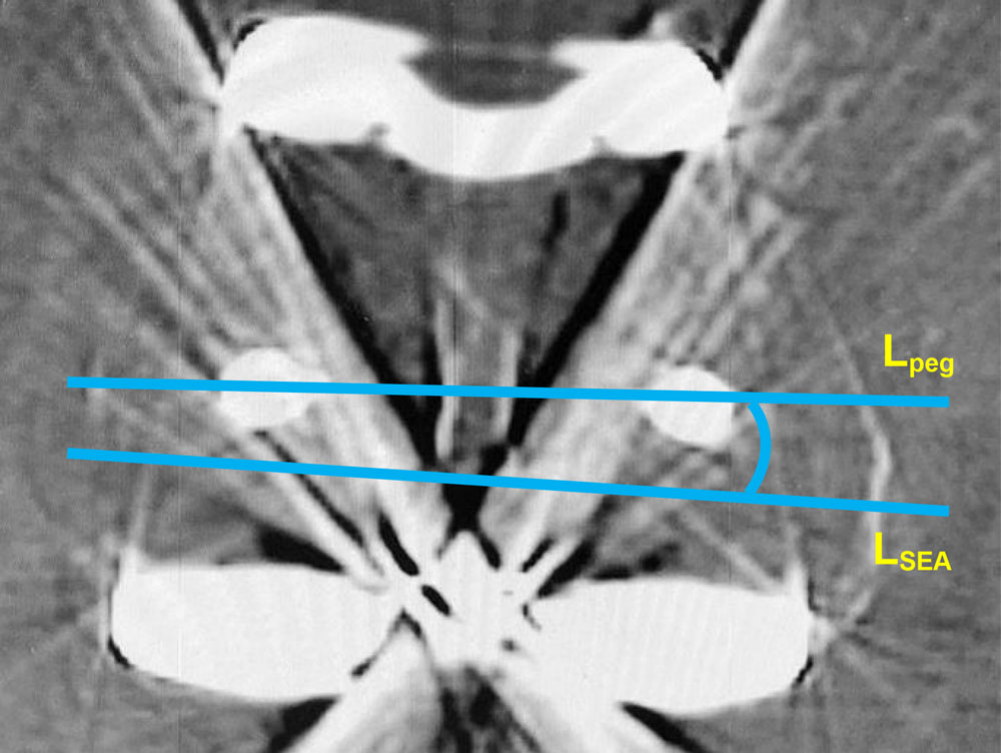
Method for measuring femoral component rotation using computed tomography. Femoral component rotation was evaluated on the axial slice where two femoral pegs and the medial and lateral epicondyles were visible. Femoral rotation was the angle between the line connecting the two pegs (*L*_peg_) and the surgical epicondylar axis (*L*_SEA_). The rotational position was classified into three groups: internal rotation from the surgical epicondylar axis (I), 0–3° external rotation (N), and >3° external rotation (E). SEA, surgical epicondylar axis.

Hip–knee–ankle (HKA) angle was measured to evaluate the whole‐leg coronal alignment [24]. An HKA angle of < 180° was considered valgus. Patella tilting angle (PTA) and patella shift (PS) were measured using the method described in previous studies [7, 21]. PTA was considered positive when the patella tilted laterally, and PS was considered positive when the patella shifted lateral to the deepest point of the femoral trochlea (Figure 3a,b). *F* (fitting) was defined as an index of the fitting depth of the patella into the trochlea and was expressed as the proportion (%) of the patellar thickness located below the anterior intercondylar line to the total thickness (Figure 3c,d). A large PTA or PS may cause patellar maltracking [7, 19], whereas a large *F* indicates patellar stability due to a deep fit into the trochlea. PTA, PS and *F* were evaluated using standardized axial radiographs in 45° knee flexion [2]. Intraclass correlation coefficient (ICC) was calculated for *F* as it was a newly defined variable. To confirm the reproducibility of *F*, 20 cases were randomly selected, and their *F* was measured two times at 2‐week intervals by two independent observers to calculate ICC. The intra‐rater ICC and inter‐rater ICC of *F* were 0.89 and 0.88, respectively, indicating good reliability [22]. The patellofemoral joint degeneration was graded using the Kellgren–Lawrence classification. Femoral component rotation was evaluated using computed tomography (Figure 4). Rotational positions were classified into three groups: internal rotation from the surgical epicondylar axis (I), 0–3° external rotation (N) and >3° external rotation (E). All radiological evaluations were performed by a single experienced orthopaedic surgeon. Because of the distinct visual characteristics of each implant on x‐ray images, blinding to the implant type was not possible during radiological assessment.

### Clinical evaluation

Data on age, sex, follow‐up (months), body mass index (BMI), diagnosis, knee extension and flexion angles (°), radiological data and patient‐reported outcome measures (PROMs) were collected. The University of California‐Los Angeles (UCLA) activity score was used to assess the physical activity of the participants [1]. The primary outcome was the Feller patellar score, used to assess patellofemoral joint function [13], whereas the secondary outcome measure was the Knee Injury and Osteoarthritis Outcome Score (KOOS), comprising five subscales [35]. All objective clinical evaluations were performed by a single skilled physical therapist who was blinded to the implant type. Evaluations were performed 1 and 2 years post‐operatively and biennially thereafter, using data from each patient's final survey for analysis.

### Data analyses

Shapiro–Wilk *W* test was used to assess normality. Continuous variables are presented as means (standard deviation) for normally distributed data and as means (standard deviation) with additional descriptive statistics [minimum, 25th percentile, median, 75th percentile, maximum] for non‐normally distributed data. Mann–Whitney *U* test was used to test the difference between the two groups. Wilcoxon signed‐rank test was used to assess differences in preoperative and post‐operative outcomes. The chi‐square test was used to determine differences in the proportions of nominal variables. Multivariate analysis of linear regression models with primary and secondary outcomes as dependent variables was performed to reduce bias. Following prior studies [6, 21, 26], the following 11 independent variables related to clinical outcomes were selected: implant type, sex, age at surgery, preoperative UCLA activity score, follow‐up (months), post‐operative BMI, post‐operative knee flexion angle (°), post‐operative PTA (°), post‐operative PS (mm), post‐operative *F* and femoral component rotation. Variance inflation factors <3 indicate no multicollinearity among the independent variables [27]. *p* Values of <0.05 were used to denote statistical significance. R (version 4.0.3: The R Foundation for Statistical Computing, Vienna, Austria) was used for all statistical analyses. A post hoc power analysis of a multiple regression model of the 11 explanatory variables, with 204 cases, an effect size of 0.28 (calculated using *R* ^2^), and an *α* of 0.01, revealed a sufficient power of 0.99.

## RESULTS

The analysis included 113 ROCC knees and 91 LCS‐RP knees. The LCS group showed a significantly longer follow‐up (*p* < 0.001), whereas no significant differences were found between the groups concerning age, sex, diagnosis and the degree of patellofemoral joint degeneration (n.s., Table 1). All factors, except for ROCC knee flexion, demonstrated significant post‐operative improvement. However, there were no significant differences between the groups for any of the factors (n.s., Table 2).

**Table 1:** Demographic data of the 113 knees of the ROCC group and 91 knees of the LCS group.^[Link]^, ^a^

Factor	ROCC (113 knees)	LCS (91 knees)	*p*
Female: % (*n*)	73.5 (83)	84.6 (77)	0.06^b^
Age at surgery (years)	73.5 (7.6) [52, 68, 75, 79, 92] c	71.1 (9.7) d	0.06^e^
Post‐operative age (years)	75.5 (7.6) d	75.3 (9.7) [48, 70, 75, 82, 95] c	0.06^e^
Follow‐up (months)	23.6 (9.4) [12, 13, 24, 28, 48] c	49.3 (28.4) [12, 24, 47, 76, 108] c	**<0.001** e
Post‐operative BMI (kg/m^2^)	26.0 (4.3) [17.9, 22.7, 25.3, 28.9, 37.5] c	25.6 (5.0) [17.3, 21.7, 24.9, 28.3, 41.1] c	0.31^e^
Diagnosis: % (*n*)	Osteoarthritis: 85.8 (97), osteonecrosis: 6.2 (7), rheumatoid arthritis: 8.0 (9)	Osteoarthritis: 80.2 (73), osteonecrosis: 5.5 (5), rheumatoid arthritis: 14.3 (13)	0.38^b^
Kellgren–Lawrence grade of patello‐femoral joint: % (*n*)	I: 15.0 (17), II: 44.2 (50), III: 29.2 (33), IV: 11.5 (13)	I: 14.2 (13), II: 45.1 (41), III: 28.6 (26), IV: 12.1 (11)	0.99^b^

*Note*: *p* Values <0.05 are in bold.

Abbreviations: BMI, body mass index; LCS, low contact stress; ROCC, rotating concave–convex.

^a^ Continuous variables are means (standard deviation) for normally distributed data and means (standard deviation) [minimum, 25th percentile, median, 75th percentile, maximum] for non‐normally distributed data.

^b^ Chi‐square test.

^c^ Non‐normally distributed data.

^d^ Normally distributed data.

^e^ Mann–Whitney *U* test.

**Table 2:** Preoperative and post‐operative knee extension–flexion angle and UCLA scores.^[Link]^, ^a^

Factor	ROCC (113 knees)	LCS (91 knees)	*p*
Preoperative knee extension angle (°) b	7.2 (8.2) [−15, 0, 5, 10, 30] c	7.8 (8.9) [0, 0, 5, 10, 30] c	0.94^d^
Post‐operative knee extension angle (°)	0.6 (1.9) [0, 0, 0, 0, 10] c	0.3 (1.5) [0, 0, 0, 0, 10] c	0.27^d^
*p*	**<0.0001** e	**<0.0001** e	
Preoperative knee flexion angle (°)	120.9 (17.1) [40, 115, 125, 130, 145] c	120.5 (16.7) [45, 110, 125, 135, 145] c	0.87^d^
Post‐operative knee flexion angle (°)	122.9 (14.2) [65, 120, 125, 130, 150] c	125.2 (12.2) [100, 120, 125, 135, 150] c	0.50^d^
*p*	0.08^e^	**0.006** e	
Preoperative UCLA score	4.2 (1.9) [1, 2, 4, 6, 10] c	4.0 (2.0) [1, 2, 4, 5, 10] c	0.29^d^
Post‐operative UCLA score	5.0 (1.9) [1, 4, 6, 10] c	5.0 (2.0) [1, 4, 6, 10] c	0.56^d^
*p*	**<0.0001** e	**<0.0001** e	

*Note*: *p* Values <0.05 are in bold.

Abbreviations: BMI, body mass index; LCS, low contact stress; ROCC, rotating concave–convex; UCLA, University of California‐Los Angeles.

^a^ Continuous variables are means (standard deviation) for normally distributed data and means (standard deviation) [minimum, 25th percentile, median, 75th percentile, maximum] for non‐normally distributed data.

^b^ The extension angle is expressed as negative for hyperextension.

^c^ Non‐normally distributed data.

^d^ Mann–Whitney *U* test.

^e^ Wilcoxon signed‐rank test.

Preoperative HKA did not differ between the groups and was significantly corrected in both (*p* < 0.0001). Post‐operatively, the ROCC group had a median varus alignment of 1.1° (*p* = 0.02). Patellar alignment and clinical outcomes were the primary focus of this study. Preoperative PTA, PS and *F* were not significantly different between the groups (n.s.). Post‐operatively, the ROCC group exhibited more horizontal and centralized patellar positioning, with a deeper fit into the trochlea compared to the LCS group (all *p* < 0.0001, details in Table 3), suggesting significantly better patellar alignment in the ROCC group.

**Table 3:** Preoperative and post‐operative radiological parameters.^[Link]^, ^a^

Factor	ROCC (113 knees)	LCS (91 knees)	*p*
Preoperative HKA (°)	189.3 (7.1) b	188.1 (7.2) [168.2, 183.5, 187.8, 188.9, 194] c	0.12^d^
Post‐operative HKA (°)	180.9 (2.8) [173.1, 179.1, 181.2, 183.6, 188.2] c	179.8 (2.7) [174.0, 178.1, 179.9, 182.5, 187.2] c	**0.02** d
*p*	**<0.0001** e	**<0.0001** e	
Preoperative PTA (°)	6.2 (3.9) [−0.7, 3.2, 5.6, 8.5, 17.9] c	6.2 (4.1) [−1.6, 3.5, 5.8, 7.9, 26.0] c	1.00^d^
Post‐operative PTA (°)	0.3 (3.2) [−6.1, −1.5, −0.4, 1.8, 16.1] c	4.3 (2.9) [−0.6, 2.1, 3.6, 6.0, 12.6] c	**<0.001** d
*p*	**<0.0001** e	**<0.0001** e	
Preoperative PS (mm)	0.9 (2.4) [−5.0, −0.7, 1.3, 2.3, 7.8] c	1.0 (2.7) [−3.2, −0.9, 1.1, 2.1, 14.6] c	0.46^d^
Post‐operative PS (mm)	0.5 (1.8) [−3.2, 0, 0, 1.4, 6.3] c	2.0 (2.5) [−1.8, 0, 1.4, 3.2, 10.5] c	**<0.001** d
*p*	**0.04** e	**0.002** e	
Preoperative *F* (%) f	13.2 (10.1) [−15.6, 8.8, 12.5, 18.3, 70.1] c	13.0 (7.7) [−12.3, 10.4, 13.8, 17.0, 29.5] c	0.68^d^
Post‐operative *F* (%)	29.5 (8.1) [10.3, 25.2, 29.1, 34.2, 56.5] c	21.4 (6.5) [−3.1, 17.3, 21.6, 25.4, 40.4] c	**<0.001** d
*p*	**<0.0001** e	**<0.0001** e	
Rotation of femoral component from SEA (°)	0.9 (1.5) [−3.3, 0.1, 0.7, 1.6, 7.0] c	0.8 (1.7) [−3.9, 0, 0.8, 2.0, 5.1] c	0.89^d^
Femoral rotation group: % (*n*)	I: 16.2 (17), N: 78.1 (82), E: 5.7 (6)	I: 20.7 (17), N: 70.7 (58), E: 8.5 (7)	0.52^g^

*Note*: *p* Values <0.05 are in bold.

Abbreviations: E, external rotation group; HKA, hip–knee–ankle angle; I, internal rotation group; LCS, low contact stress; N, neutral group; PS, patella shift; PTA, patella tilting angle; ROCC, rotating concave–convex; SEA, surgical epicondylar axis.

^a^ Continuous variables are means (standard deviation) for normally distributed data and means (standard deviation) [minimum, 25th percentile, median, 75th percentile, maximum] for non‐normally distributed data.

^b^ Normally distributed data.

^c^ Non‐normally distributed data.

^d^ Mann–Whitney U‐test.

^e^ Wilcoxon signed‐rank test.

^f^ F is expressed negative when the central ridge of the patella position is shallower than the anterior intercondylar line.

^g^ Chi‐square test.

Clinical outcomes also favoured the ROCC group, with significantly higher KOOS‐Symptom scores (*p* = 0.04, Table 4). Multiple regression analyses revealed that ROCC selection positively influenced primary (patellar score) and secondary (KOOS subscales) outcomes. Additional regression findings showed that preoperative UCLA scores positively impacted patellar scores, KOOS‐Pain and KOOS‐ADL. Post‐operative knee flexion angle positively affected all KOOS subscales, whereas age at surgery negatively impacted KOOS‐ADL. Interestingly, despite no effects of PTA and PS on outcomes, *F* showed a negative effect on KOOS‐Sports (*β* = −0.222, *p* = 0.04, Table 5). Detailed results are presented in Table S1.

**Table 4:** Post‐operative PROMs used to assess primary (patellar score) and secondary (KOOS) clinical outcomes.^[Link]^, ^a^

PROMs	ROCC (113 knees)	LCS (91 knees)	*p*
Patellar score	26.9 (3.7) [14, 25.0, 29, 30, 30] b	25.9 (3.9) [15, 23.5, 27, 29, 30] b	0.059^c^
KOOS			
Pain	86.9 (16.4) [30.6, 79.9, 93.1, 100, 100] b	84.5 (16.1) [30.6, 78.5, 88.9, 97.2, 100] b	0.09^c^
Symptom	86.8 (11.9) [53.8, 81.3, 89.3, 96.4, 100] b	83.0 (13.9) [21.4, 75.0, 85.7, 92.9, 100] b	**0.04** c
ADL	82.9 (16.0) [30.9, 76.1, 86.8, 94.1, 100] b	80.4 (15.9) [23.5, 72.1, 83.8, 92.6, 100] b	0.14^c^
Sports	53.3 (30.9) [0, 30, 55, 80, 100] b	45.8 (27.3) [0, 25, 45, 65, 100] b	0.053^c^
QOL	65.3 (27.7) [0, 50, 71.9, 81.3, 100] b	61.1 (28.6) [0, 37.5, 68.8, 87.5, 100] b	0.26^c^

*Note*: *p* Values <0.05 are in bold.

Abbreviations: ADL, activity of daily living; KOOS, knee injury and osteoarthritis outcome score; LCS, low contact stress; PROM, patient‐reported outcome measure; QOL, quality of life; ROCC, rotating concave–convex.

^a^ Continuous variables are means (standard deviation) for normally distributed data and means (standard deviation) [minimum, 25th percentile, median, 75th percentile, maximum] for non‐normally distributed data.

^b^ Non‐normally distributed data.

^c^ Mann–Whitney *U* test.

**Table 5:** Significant explanatory variables for multiple regression analysis with primary (patellar score) and secondary (KOOS) outcomes as response variables.

Response variable	Significant explanatory variables	Estimate (95% CI)	Standard error	*β*	*t*‐Value	*p*	VIF
Patellar score	Implant [ROCC]	2.63 (0.87–4.39)	0.89	0.343	2.96	**0.004**	2.26
	Preoperative UCLA score	0.51 (0.15–0.86)	0.18	0.239	2.84	**0.005**	1.19
	Follow‐up (months)	0.06 (0.03–0.09)	0.02	0.381	3.70	**0.0003**	1.78
KOOS pain	Implant [ROCC]	7.93 (1.45–14.4)	3.28	0.241	2.42	**0.02**	2.00
	Preoperative UCLA score	1.83 (0.53–3.12)	0.66	0.214	2.79	**0.006**	1.18
	Follow‐up (months)	0.23 (0.1–0.35)	0.06	0.328	3.69	**0.0003**	1.59
	Post‐operative knee flexion angle (°)	0.25 (0.07–0.42)	0.09	0.201	2.79	**0.006**	1.05
KOOS symptom	Implant [ROCC]	8.04 (2.98–13.09)	2.56	0.307	3.14	**0.002**	2.00
	Follow‐up (months)	0.15 (0.05–0.24)	0.05	0.265	3.05	**0.003**	1.59
	Post‐operative knee flexion angle (°)	0.3 (0.16–0.43)	0.07	0.304	4.31	**<0.0001**	1.05
KOOS ADL	Implant [ROCC]	8.06 (1.77–14.35)	3.19	0.247	2.53	**0.01**	2.00
	Age at surgery (years)	−0.34 (−0.64 to −0.04)	0.15	−0.179	−2.22	**0.03**	1.36
	Preoperative UCLA score	1.62 (0.37–2.88)	0.64	0.192	2.55	**0.01**	1.18
	Follow‐up (months)	0.19 (0.07–0.3)	0.06	0.274	3.15	**0.002**	1.59
	Post‐operative knee flexion angle (°)	0.32 (0.16–0.49)	0.09	0.267	3.79	**<0.0001**	1.05
KOOS sports	Implant [ROCC]	20.67 (9.05–32.3)	5.89	0.344	3.51	**0.0006**	2.00
	Follow‐up (months)	0.33 (0.11–0.55)	0.11	0.263	3.02	**0.003**	1.59
	Post‐operative knee flexion angle (°)	0.32 (0.01–0.63)	0.16	0.144	2.04	**0.04**	1.05
	Post‐operative *F* (%)	−0.78 (−1.51 to 0.05)	0.37	−0.222	−2.10	**0.04**	2.34
	Femoral rotation group [*N*]	11.82 (0.85–22.79)	5.56	0.172	2.13	**0.03**	1.36
KOOS QOL	Implant [ROCC]	20.07 (9.29–30.85)	5.46	0.355	3.68	**0.0003**	2.00
	Follow‐up (months)	0.41 (0.21–0.61)	0.10	0.347	4.04	**0.0001**	1.59
	Post‐operative knee flexion angle (°)	0.54 (0.25–0.83)	0.15	0.258	3.69	**0.0003**	1.05

*Note*: *p* values < 0.05 are in bold.

Abbreviations: ADL, activity of daily living; β, standardized partial regression coefficient; BMI, body mass index; CI, confidence interval; KOOS, knee injury and osteoarthritis outcome score; N, neutral; PS, patella shift; PTA, patella tilting angle; QOL, quality of life; ROCC, rotating concave–convex; UCLA, University of California‐Los Angeles; VIF, variance inflation factor.

## DISCUSSION

The main findings of the current study are that the native patella in the ROCC group was more horizontally, centrally, and deeply fitted than in the LCS group. Furthermore, the clinical outcomes of ROCC, including patellofemoral function, outperformed those of LCS‐RP, confirming our hypothesis.

Trochlear designs with a higher lateral anterior condyle height (ACH) or medial and lateral ACH have previously been associated with a reduced PTA [25]. Thus, the deeper trochlea and higher lateral and medial ACH in ROCC likely contributed to its smaller PTA compared to LCS.

Although femoral component rotation has been correlated with post‐operative PTA [9], it was similar between the two groups in our study, suggesting that the observed differences in patellar alignment are primarily because of the femoral component geometry rather than rotational positioning. Optimal results have been reported with a femoral component rotation of 2–5° to the femoral posterior condyle axis [28], which aligns with the result of our study. A previous study reported similar patellar scores in groups with varying post‐operative PTA/PS [21], which agrees with our findings that post‐operative PTA and PS were not significant independent variables for patellar scores.

In the multiple regression analyses, the consistent positive effect of ROCC implant selection on primary and secondary outcomes, even after controlling for other variables, supports the overall superiority of this design in our cohort. Additionally, there was no difference in the degree of preoperative patellofemoral degeneration between the groups. The ROCC group had a median patellar score of 29 points, compared with 27 points for the LCS‐RP group. This aligns with previous studies reporting mean or median scores of 23.8–28 points for LCS‐RP with the native patella at long‐term follow‐up [18, 29, 32]. A previous study indicating that a deep and long trochlea decreases patellar morbidity supports the results of our study [28]. The superior outcomes of ROCC can be attributed to several design features: (1) ROCC demonstrated greater conformity at 0–60° of knee flexion, whereas LCS‐RP excelled at 0–30° [10, 41]. (2) The saddle‐shaped insert provides stability by guiding sagittal motion and offering soft endpoints in the mediolateral and anteroposterior directions [5]. These features likely contribute to ROCC's ability to reduce mid‐flexion instability, which can cause poor outcomes and patient dissatisfaction in up to 20% of TKA cases [30].

A previous study found that elevated patellofemoral pressure due to increased ACH negatively impacted the advanced activity subscales of the Knee Society Score‐2011 [38]. In our study, a larger *F* negatively affected KOOS‐Sports, particularly for activities like kneeling and squatting. This suggests a potential trade‐off between patellar deep‐fitting and certain high‐demand activities. A relatively larger ACH due to a larger F could also compress the peripatellar retinaculum and synovium [28], where many free nerve endings and fibres are located. However, ROCC's overall design appears to mitigate these potential drawbacks. Its finer anteroposterior femoral size pitch (2 mm vs. LCS's 3.1–5.4 mm) facilitates flush cutting of the anterior femoral surface, potentially reducing ACH while maintaining posterior condylar offset. The saddle‐shaped insert may further optimize patellar biomechanics. These features likely contribute to ROCC's superior overall clinical outcomes despite the larger *F*.

Unlike a previous study that reported significantly better Oxford Knee Scores for ROCC than for fixed‐bearing PCL‐retained TKA (mean 38 vs. 34 points, *p* = 0.047) [39], our study directly compared ROCC with LCS‐RP. This comparison is particularly valuable as these designs have similar fundamental principles but different specific features of their trochlear and insert designs. Our direct comparison revealed specific advantages of ROCC concerning patellar alignment and clinical outcomes.

Our study's findings regarding preoperative UCLA scores, follow‐up duration, surgical age and post‐operative knee flexion angle align with previous studies [31, 33, 34, 40]. These results underscore the importance of preoperative patient condition, rehabilitation time and post‐operative range of motion in determining TKA outcomes. The post‐operative HKA of the ROCC group was ~1.1° varus compared with the LCS group. Both groups remained within the acceptable range [24], so this discrepancy likely had minimal impact on clinical outcomes [15].

Our study offers novel contributions to the literature on TKA designs. First, the superior short‐term clinical outcomes and improved patellar stability observed with the ROCC have important implications for TKA practice, suggesting that this design may be especially beneficial for patients undergoing TKA with patellar‐nonresurfacing, potentially improving overall knee function. Second, our study introduces a new radiographic parameter *F* as an indicator of patellar fitting depth, which could serve as a useful tool for future research and clinical assessment of patellofemoral articulation in TKA.

This study has several limitations. First, the follow‐up period was short (median: ROCC: 24 months, LCS‐RP: 47 months, overall: 25 months). In our cohort, no patients required secondary patellar resurfacing. However, ROCC had an incidence of 3.4% (6/174 knees) for secondary patellar resurfacing after a mean follow‐up of 7.5 years [5], whereas LCS‐RP has an incidence of 0.4% (2/500 knees) after a mean follow‐up of 18.1 years [29]. The patient specification for the requirement of secondary patellar resurfacing and surgeons' decisions in each study remained unclear, thus making a direct comparison unviable. Further follow‐up in our cohort is warranted to evaluate the robust outcomes of patellar‐nonresurfacing ROCC versus LCS‐RP. Second, the retrospective design of this study might have led to selection bias due to the lack of randomization. The learning curve might influence selection bias in ROCC/LCS‐RP usage due to frequent ROCC later in the study period. Third, modest sample size and potential confounding variables might have affected the outcomes. Fourth, the Feller score would be too simple to accurately reveal the differences between the groups. Fifth, assessments of patellar length and height on lateral radiographs were not performed. Sixth, despite the significant differences in post‐operative patellar alignment between ROCC and LCS, their direct impact on clinical outcomes was unclear. Finally, although this study considered the theoretical advantages of ROCC's geometry, only patellar alignment was assessed. Nonetheless, ROCC's structural stability likely positively influenced primary and secondary outcomes. Further research comprising a larger cohort and longer‐term follow‐up to confirm the factors contributing to the clinical outcomes of TKA, including mid‐flexion stability, might reveal the structural stability of ROCC.

## CONCLUSIONS

At short‐term follow‐up, ROCC stabilized the native patella further horizontally, centrally, and deeply into its deeper and longer trochlea, thus outperforming the established LCS‐RP design clinically. Although long‐term studies are needed, the structural improvements of the ROCC may offer tangible benefits for patients. Orthopaedic surgeons should consider these potential advantages while selecting TKA models, especially those featuring cruciate‐sacrificing RP mechanisms in patellar‐nonresurfacing procedures.

## AUTHOR CONTRIBUTIONS

Masafumi Itoh conceived and designed the analysis, performed the analysis and wrote the manuscript. Ken Okazaki supervised the research design. All authors performed data collection and approved the final manuscript.

## FUNDING INFORMATION

This research did not receive any specific grant from funding agencies in the public, commercial or not‐for‐profit sectors.

## CONFLICT OF INTEREST STATEMENT

The authors declare no conflict of interest.

## ETHICS STATEMENT

This retrospective cohort study was approved by the Ethics Committee of Tokyo Women's Medical University (approval number: 4578) and conducted in accordance with the ethical standards of the Declaration of Helsinki. Informed consent was obtained from all patients.

## Supporting information

Supporting information.

## Data Availability

The data sets used and/or analyzed during the current study are available from the corresponding author upon reasonable request.
