# Supplementary material for: Saddle‐shaped rotating knee prosthesis outperforms low contact stress knee: A short‐term retrospective comparative study
Source: J Exp Orthop. 2024 Dec 2;11(4):e70072. doi: 10.1002/jeo2.70072 (PMC11610624; doi:10.1002/jeo2.70072)
Supplement: Supplementary file 1 — Supporting information. [file JEO2-11-e70072-s001.docx]

*Journal of Experimental Orthopaedics*

**Saddle-shaped Rotating Knee Prosthesis Outperforms Low Contact Stress Knee: A Short-term Retrospective Comparative Study**

Supplementary table 1. Detail of multiple regression analyses with primary and secondary outcomes as the response variables

|  | **Factor** | **Estimate (95% CI)** | **Standard Error** | **β** | **t-value** | ***P*-value** | **VIF** |
| --- | --- | --- | --- | --- | --- | --- | --- |
| Patellar score | Model (*p*-value = **0.0008**, R = 0.47) | | | | | | |
|  | (Intercept) | 12.4 (1.6–23.2) | 5.46 |  | 2.27 | **0.02** |  |
|  | Implant [ROCC] | 2.63 (0.87–4.39) | 0.89 | 0.343 | 2.96 | **0.004** | 2.26 |
|  | Sex [male] | 0.64 (−0.94–2.14) | 0.78 | 0.067 | 0.77 | 0.44 | 1.27 |
|  | Age at surgery (year) | 0.08 (−0.01–0.17) | 0.05 | 0.165 | 1.77 | 0.08 | 1.45 |
|  | Preoperative UCLA score | 0.51 (0.15–0.86) | 0.18 | 0.239 | 2.84 | **0.005** | 1.19 |
|  | Follow-up (month) | 0.06 (0.03–0.09) | 0.02 | 0.381 | 3.70 | **0.0003** | 1.78 |
|  | Postoperative BMI | −0.05 (−0.19–0.1) | 0.07 | −0.060 | −0.66 | 0.51 | 1.41 |
|  | Postoperative knee flexion angle (°) | 0.04 (0–0.08) | 0.02 | 0.155 | 1.92 | 0.06 | 1.09 |
|  | Postoperative PTA (°) | −0.16 (−0.41–0.09) | 0.13 | −0.153 | −1.25 | 0.21 | 2.52 |
|  | Postoperative PS (mm) | 0.27 (−0.08–0.61) | 0.17 | 0.163 | 1.52 | 0.13 | 1.91 |
|  | Postoperative *F* (%) | −0.06 (−0.17–0.05) | 0.06 | −0.137 | −1.13 | 0.26 | 2.50 |
|  | Femoral rotation group [N] | −0.06 (−1.59–1.48) | 0.77 | −0.007 | −0.07 | 0.94 | 1.36 |
|  | Femoral rotation group [E] | 0.71 (−1.98–3.39) | 1.36 | 0.045 | 0.52 | 0.60 | 1.28 |
| KOOS Pain | Model (*p*-value = **0.002**, R = 0.40) | | | | | | |
|  | (Intercept) | 14.79 (−26.1–55.69) | 20.72 |  | 0.71 | 0.48 |  |
|  | Implant [ROCC] | 7.93 (1.45–14.4) | 3.28 | 0.241 | 2.42 | **0.02** | 2.00 |
|  | Sex [male] | −0.92 (−7.08–5.24) | 3.12 | −0.023 | −0.30 | 0.77 | 1.25 |
|  | Age at surgery (year) | 0.14 (−0.17–0.45) | 0.16 | 0.074 | 0.89 | 0.37 | 1.36 |
|  | Preoperative UCLA score | 1.83 (0.53–3.12) | 0.66 | 0.214 | 2.79 | **0.006** | 1.18 |
|  | Follow-up (month) | 0.23 (0.1–0.35) | 0.06 | 0.328 | 3.69 | **0.0003** | 1.59 |
|  | Postoperative BMI | 0.27 (−0.28–0.82) | 0.28 | 0.076 | 0.97 | 0.34 | 1.24 |
|  | Postoperative knee flexion angle (°) | 0.25 (0.07–0.42) | 0.09 | 0.201 | 2.79 | **0.006** | 1.05 |
|  | Postoperative PTA (°) | 0.03 (−0.94–0.99) | 0.49 | 0.006 | 0.05 | 0.96 | 2.41 |
|  | Postoperative PS (mm) | −0.2 (−1.45–1.06) | 0.64 | −0.028 | −0.31 | 0.75 | 1.61 |
|  | Postoperative *F* (%) | 0.01 (−0.39–0.42) | 0.21 | 0.007 | 0.06 | 0.95 | 2.34 |
|  | Femoral rotation group [N] | 4.35 (−1.76–10.46) | 3.10 | 0.116 | 1.40 | 0.16 | 1.36 |
|  | Femoral rotation group [E] | 9.56 (−0.83–19.95) | 5.26 | 0.146 | 1.82 | 0.07 | 1.30 |
| KOOS Symptom | Model (*p*-value = **0.0001**, R = 0.45) | | | | | | |
|  | (Intercept) | 26.42 (−5.49–58.33) | 16.16 |  | 1.63 | 0.10 |  |
|  | Implant [ROCC] | 8.04 (2.98–13.09) | 2.56 | 0.307 | 3.14 | **0.002** | 2.00 |
|  | Sex [male] | 1.01 (−3.79–5.82) | 2.43 | 0.032 | 0.42 | 0.68 | 1.25 |
|  | Age at surgery (year) | 0.01 (−0.24–0.25) | 0.12 | 0.003 | 0.04 | 0.97 | 1.36 |
|  | Preoperative UCLA score | 0.52 (−0.49–1.53) | 0.51 | 0.077 | 1.02 | 0.31 | 1.18 |
|  | Follow-up (month) | 0.15 (0.05–0.24) | 0.05 | 0.265 | 3.05 | **0.003** | 1.59 |
|  | Postoperative BMI | 0.13 (−0.3–0.55) | 0.22 | 0.044 | 0.58 | 0.56 | 1.24 |
|  | Postoperative knee flexion angle (°) | 0.3 (0.16–0.43) | 0.07 | 0.304 | 4.31 | **<0.0001** | 1.05 |
|  | Postoperative PTA (°) | 0.32 (−0.44–1.07) | 0.38 | 0.089 | 0.83 | 0.41 | 2.41 |
|  | Postoperative PS (mm) | −0.33 (−1.3–0.65) | 0.50 | −0.058 | −0.66 | 0.51 | 1.61 |
|  | Postoperative *F* (%) | 0.15 (−0.17–0.47) | 0.16 | 0.099 | 0.94 | 0.35 | 2.34 |
|  | Femoral rotation group [N] | 2.27 (−2.5–7.04) | 2.42 | 0.075 | 0.94 | 0.35 | 1.36 |
|  | Femoral rotation group [E] | 6.72 (−1.38–14.83) | 4.11 | 0.129 | 1.64 | 0.10 | 1.30 |
| KOOS ADL | Model (*p*-value = **0.0001**, R = 0.45) | | | | | | |
|  | (Intercept) | 54.97 (15.27–94.68) | 20.11 |  | 2.73 | 0.01 |  |
|  | Implant [ROCC] | 8.06 (1.77–14.35) | 3.19 | 0.247 | 2.53 | **0.01** | 2.00 |
|  | Sex [male] | −1.9 (−7.88–4.08) | 3.03 | −0.048 | −0.63 | 0.53 | 1.25 |
|  | Age at surgery (year) | −0.34 (−0.64–−0.04) | 0.15 | −0.179 | −2.22 | **0.03** | 1.36 |
|  | Preoperative UCLA score | 1.62 (0.37–2.88) | 0.64 | 0.192 | 2.55 | **0.01** | 1.18 |
|  | Follow-up (month) | 0.19 (0.07–0.3) | 0.06 | 0.274 | 3.15 | **0.002** | 1.59 |
|  | Postoperative BMI | −0.23 (−0.76–0.31) | 0.27 | −0.064 | −0.83 | 0.41 | 1.24 |
|  | Postoperative knee flexion angle (°) | 0.32 (0.16–0.49) | 0.09 | 0.267 | 3.79 | **<0.0001** | 1.05 |
|  | Postoperative PTA (°) | −0.44 (−1.37–0.5) | 0.48 | −0.098 | −0.92 | 0.36 | 2.41 |
|  | Postoperative PS (mm) | −0.31 (−1.53–0.91) | 0.62 | −0.044 | −0.50 | 0.62 | 1.61 |
|  | Postoperative *F* (%) | −0.1 (−0.5–0.29) | 0.20 | −0.053 | −0.51 | 0.61 | 2.34 |
|  | Femoral rotation group [N] | 3.47 (−2.47–9.4) | 3.01 | 0.093 | 1.15 | 0.25 | 1.36 |
|  | Femoral rotation group [E] | 6.67 (−3.42–16.75) | 5.11 | 0.103 | 1.30 | 0.19 | 1.30 |
| KOOS Sports | Model (*p*-value = **0.0002**, R = 0.44) | | | | | | |
|  | (Intercept) | 28.03 (−45.36–101.42) | 37.18 |  | 0.75 | 0.45 |  |
|  | Implant [ROCC] | 20.67 (9.05–32.3) | 5.89 | 0.344 | 3.51 | **0.0006** | 2.00 |
|  | Sex [male] | 10.32 (−0.73–21.37) | 5.60 | 0.143 | 1.84 | 0.07 | 1.25 |
|  | Age at surgery (year) | −0.21 (−0.77–0.34) | 0.28 | −0.061 | −0.76 | 0.45 | 1.36 |
|  | Preoperative UCLA score | 1.56 (−0.76–3.88) | 1.18 | 0.100 | 1.33 | 0.19 | 1.18 |
|  | Follow-up (month) | 0.33 (0.11–0.55) | 0.11 | 0.263 | 3.02 | **0.003** | 1.59 |
|  | Postoperative BMI | −0.9 (−1.89–0.08) | 0.50 | −0.139 | −1.80 | 0.07 | 1.24 |
|  | Postoperative knee flexion angle (°) | 0.32 (0.01–0.63) | 0.16 | 0.144 | 2.04 | **0.04** | 1.05 |
|  | Postoperative PTA (°) | −0.21 (−1.95–1.52) | 0.88 | −0.026 | −0.24 | 0.81 | 2.41 |
|  | Postoperative PS (mm) | −0.32 (−2.58–1.93) | 1.14 | −0.025 | −0.28 | 0.78 | 1.61 |
|  | Postoperative *F* (%) | −0.78 (−1.51–−0.05) | 0.37 | −0.222 | −2.10 | **0.04** | 2.34 |
|  | Femoral rotation group [N] | 11.82 (0.85–22.79) | 5.56 | 0.172 | 2.13 | **0.03** | 1.36 |
|  | Femoral rotation group [E] | 16.44 (−2.2–35.09) | 9.44 | 0.137 | 1.74 | 0.08 | 1.30 |
| KOOS QOL | Model (*p*-value = **0.00003**, R = 0.47) | | | | | | |
|  | (Intercept) | 5.59 (−62.45–73.64) | 34.47 |  | 0.16 | 0.87 |  |
|  | Implant [ROCC] | 20.07 (9.29–30.85) | 5.46 | 0.355 | 3.68 | **0.0003** | 2.00 |
|  | Sex [male] | −9.22 (−19.47–1.03) | 5.19 | −0.136 | −1.78 | 0.08 | 1.25 |
|  | Age at surgery (year) | −0.37 (−0.88–0.15) | 0.26 | −0.112 | −1.41 | 0.16 | 1.36 |
|  | Preoperative UCLA score | 0.93 (−1.22–3.08) | 1.09 | 0.063 | 0.85 | 0.40 | 1.18 |
|  | Follow-up (month) | 0.41 (0.21–0.61) | 0.10 | 0.347 | 4.04 | **0.0001** | 1.59 |
|  | Postoperative BMI | 0.13 (−0.79–1.04) | 0.46 | 0.021 | 0.28 | 0.78 | 1.24 |
|  | Postoperative knee flexion angle (°) | 0.54 (0.25–0.83) | 0.15 | 0.258 | 3.69 | **0.0003** | 1.05 |
|  | Postoperative PTA (°) | −0.81 (−2.41–0.8) | 0.81 | −0.105 | −0.99 | 0.32 | 2.41 |
|  | Postoperative PS (mm) | −0.38 (−2.47–1.71) | 1.06 | −0.031 | −0.36 | 0.72 | 1.61 |
|  | Postoperative *F* (%) | −0.59 (−1.26–0.09) | 0.34 | −0.178 | −1.71 | 0.09 | 2.34 |
|  | Femoral rotation group [N] | 3.83 (−6.34–14) | 5.15 | 0.059 | 0.74 | 0.46 | 1.36 |
|  | Femoral rotation group [E] | 16.76 (−0.52–34.05) | 8.76 | 0.149 | 1.91 | 0.06 | 1.30 |

*P*-values <0.05 are in bold.

KOOS, knee injury and osteoarthritis outcome score; PROMs, patient-reported outcome measures; CI, confidence interval; β, standardized partial regression coefficient; VIF, variance inflation factor; R, multiple correlation coefficient; ROCC, rotating concave–convex; UCLA, University of California-Los Angeles; BMI, body mass index; PTA, patella tilting angle; PS, patella shift; N, neutral; E, external rotation; ADL, activity of daily living; QOL, quality of life
